# Impact of Drug Loading Method on Drug Release from 3D-Printed Tablets Made from Filaments Fabricated by Hot-Melt Extrusion and Impregnation Processes

**DOI:** 10.3390/pharmaceutics13101607

**Published:** 2021-10-03

**Authors:** Kasitpong Thanawuth, Lalinthip Sutthapitaksakul, Srisuda Konthong, Supakij Suttiruengwong, Kampanart Huanbutta, Crispin R. Dass, Pornsak Sriamornsak

**Affiliations:** 1 Department of Pharmaceutical Technology, Faculty of Pharmacy, Silpakorn University, Nakhon Pathom 73000, Thailand; thanawuth_k@silpakorn.edu (K.T.); sutthapitak_l@silpakorn.edu (L.S.); 2 Pharmaceutical Biopolymer Group (PBiG), Silpakorn University, Nakhon Pathom 73000, Thailand; srisuda.ann23@gmail.com (S.K.); kampanart@go.buu.ac.th (K.H.); 3 Department of Materials Science and Engineering, Faculty of Engineering and Industrial Technology, Silpakorn University, Nakhon Pathom 73000, Thailand; suttiruengwong_s@su.ac.th; 4 Faculty of Pharmaceutical Sciences, Burapha University, Chonburi 20131, Thailand; 5 Curtin Medical School, Faculty of Health Sciences, Curtin University, Perth 6845, Australia; crispin.dass@curtin.edu.au; 6 Curtin Health Innovation Research Institute, Bentley 6102, Australia; 7 Academy of Science, The Royal Society of Thailand, Bangkok 10300, Thailand

**Keywords:** 3D-printed tablet, hot-melt extrusion, impregnation, polyvinyl alcohol, indomethacin, filament

## Abstract

The purpose of this study was to investigate the impact of the drug loading method on drug release from 3D-printed tablets. Filaments comprising a poorly water-soluble model drug, indomethacin (IND), and a polymer, polyvinyl alcohol (PVA), were prepared by hot-melt extrusion (HME) and compared with IND-loaded filaments prepared with an impregnation (IMP) process. The 3D-printed tablets were fabricated using a fused deposition modeling 3D printer. The filaments and 3D printed tablets were evaluated for their physicochemical properties, swelling and matrix erosion behaviors, drug content, and drug release. Physicochemical investigations revealed no drug–excipient interaction or degradation. IND-loaded PVA filaments produced by IMP had a low drug content and a rapid drug release. Filaments produced by HME with a lower drug content released the drug faster than those with a higher drug content. The drug content and drug release of 3D-printed tablets containing IND were similar to those of the filament results. Particularly, drug release was faster in 3D-printed tablets produced with filaments with lower drug content (both by IMP and HME). The drug release of 3D-printed tablets produced from HME filaments with higher drug content was extended to 24 h due to a swelling-erosion process. This study confirmed that the drug loading method has a substantial influence on drug content, which in turn has a significant effect on drug release. The results suggest that increasing the drug content in filaments might delay drug release from 3D-printed tablets, which may be used for developing dosage forms suited for personalized medicine.

## 1. Introduction

In recent years, there has been a surge in interest in three-dimensional (3D) printing technology, which is described as a procedure for creating objects in which materials are repeatedly deposited layer by layer using a computerized system based on digital information. This technology has been used in a variety of applications, including aerospace [1], automotive [2], medical devices, tissue engineering, and pharmaceuticals [3]. The implementation of 3D printing technology in the pharmaceutical industry has received a lot of attention, and over the last decade, it has evolved into an exciting invention. Spritam^®^ (levetiracetam), the first 3D printed orodispersible tablet, was authorized by the FDA in 2015 [4]. Because of its applicability in the pharmaceutical industry, the number of research publications on 3D printing technologies has increased rapidly. These include, for example, complex drug release profiles, personalized and unique drug dosage, customizable and on-demand drug printing, and implanted drug delivery devices [5]. Binder jetting, vat photopolymerization, powder bed fusion, material jetting, material extrusion, directed energy deposition, and sheet lamination are a few examples of 3D printing processes [6]. Among the above-mentioned types, material extrusion, fused deposition modeling (FDM) is extensively utilized because of its benefits. FDM has the advantages of being simple to operate, portable, and low-cost [7].

FDM refers to a 3D printing technique that employs a continuous filament of a thermoplastic substance. A thermoplastic filament is fed continuously into a tiny heated chamber, where it melts and becomes a very viscous fluid. The melt is then extruded via a nozzle and deposited layer by layer on a heated table, according to a pattern determined by the printer control software to imitate the desired shape of the item, which may be given as a CAD file, generally in stereo lithography interface format (STL) [8]. FDM filament materials include acrylonitrile butadiene styrene, polylactic acid, polyethylene terephthalate glycol, polyethylene terephthalate, thermoplastic polyurethane, and aliphatic polyamides (nylon). However, typical filament materials utilized in pharmaceutical applications include crosslinked polyacrylic acid, polycaprolactone, polylactic acid, polyvinylpyrrolidone, polyvinyl alcohol (PVA), and others. PVA, a non-toxic and biodegradable polymer used in the food, medical, and pharmaceutical industries, has proven to be an excellent solid dispersion carrier for enhanced drug absorption [9]. PVA has gained a lot of attention in regard to FDM 3D printing dosage forms because of its advantages including biocompatibility, biodegradability, and water-soluble characteristics. Furthermore, because the melting temperature of this polymer ranges from 170 °C to 228 °C, depending on the degree of hydrolysis of acetate groups [10], it remains stable during the printing process.

The filament used in FDM is capable of being loaded with variable concentrations of drug. Generally, the drug is incorporated into the filament prior to the printing process. Common techniques to load drugs into the filaments include impregnation (IMP) and hot-melt extrusion (HME). The IMP process is easy to operate and does not require any heating. The IMP process is based mostly on passive diffusion, implying that saturated or highly concentrated drug solutions are necessary for incorporation. The majority of the drug in the solution is partly absorbed at the surface of the filaments [11]. This method was used to incorporate multiple drugs into commercially available filaments by immersing them in a saturated alcoholic solution containing drug for an appropriate period of time. The amount of drug loaded has been found to vary. Several authors have reported on the IMP process for drug loading into the filaments for 3D printing [10]. However, because of the low drug loading (approximately 2% *w*/*w*), it is only appropriate for drugs with a therapeutic effect at low doses [12]. Unlike IMP, HME is a technique that combines a thermoplastic polymer with a drug at a high temperature, generally above the melting point of the polymer and drug. The drug and polymer are fed into the hopper, mixed in the screw, and then extruded from the nozzle as a filament with a diameter suitable for FDM 3D printing machines [13]. HME has been utilized to mask drug bitterness, enhance drug dissolution, and enable prolonged drug release [14]. It has been reported that HME can be used to produce polymer filaments for FDM 3D printing [15]. 

Previously, most research assessed the internal structure, size, and geometry of 3D-printed dosage forms to see how these factors impact drug release from 3D-printed tablets. To the best of our knowledge, no research has been published on the effect of the technique used to load drugs into filament on the drug content of filaments and 3D-printed tablets, and its impact on drug release. Therefore, this study was aimed at examining the impact of drug loading methods, HME and IMP, on drug content and drug release from 3D-printed tablets produced by FDM 3D printing. 

## 2. Materials and Methods

Indomethacin (IND), a poorly water-soluble drug categorized as a BCS class II drug, was used as a model drug and was purchased from P.C. Drug Center Co., Ltd., Bangkok, Thailand. PVA granules (Mowiflex^®^ C17, Kuraray, Tokyo, Japan) were chosen as a polymer for the extrusion process. A commercial PVA filament (CCTREE, Shenzhen Primes Technology Co., Ltd., Shenzhen, China) was used for FDM 3D printing (1.75-mm diameter, print temperature 190–220 °C). Other chemicals, such as organic solvents and buffering agents, were of reagent grade or analytical grade.

### 2.1. Drug-Loaded Filament Preparation

#### 2.1.1. HME Method

The PVA granules were pulverized into powder using a microfine grinder drive (IKA-Werke GmbH & Co., KG, Staufen, Germany). Following that, 30% *w*/*w* of IND and PVA powder were mixed for 5 min. The filament extruder utilized was a single-screw extruder (model Wellzoom^TM^ C desktop extruder, Shenzhen Mistar Technology Co., Ltd., Shenzhen, China). The mixture was extruded through a nozzle (1.75 mm in diameter) at 180 °C using a screw speed of 12 rpm. The IND-loaded filament was then placed in a desiccator for storage. Meanwhile, PVA filaments with a low drug loading, approximately 5% *w*/*w*, and no IND were produced using the same procedure described above.

#### 2.1.2. IMP Method

PVA commercial filament and PVA filament prepared by HME (3-m long) were soaked in a saturated solution of IND in methanol at 40 °C for 24 h in a shaker-incubator (model ES-20, Biosan, Riga, Latvia). The filaments were then dried in an oven at 60 °C until they reached a steady weight. Finally, the IND-loaded filaments were kept in a desiccator.

### 2.2. FDM 3D Printing Process

The 3D-printed tablet was designed with a cylindrical shape, a diameter of 7.00 mm and a thickness of 4.00 mm using AutoCAD 2018 software (Autodesk Inc., San Rafael, CA, USA). Then, the model was saved in stereolithography (STL) format and imported into the 3D printer software, FlashPrint software version 3.28.0 (Zhejiang Flashforge 3D Technology Co., Ltd., Zhejiang, China). The 3D-printed tablets were produced using Flashforge Creator Pro (Zhejiang Flashforge 3D Technology Co., Ltd., Zhejiang, China). Printing temperatures of 200 °C and 190 °C were used for the tablets prepared from IND-loaded filaments using HME and IMP, respectively. The other printing process settings were: a bed temperature of 60 °C, a printing speed of 20 mm/s, a moving speed of 70 mm/s, a 100% infill density with a line pattern, and a layer thickness of 0.12 mm. 

### 2.3. Characterization of Filaments and/or 3D-Printed Tablets

#### 2.3.1. Differential Scanning Calorimetry (DSC)

The DSC 8000 (PerkinElmer, Waltham, MA, USA) was used to analyze the thermal characteristics of the materials, including IND, PVA powder, physical mixtures, PVA commercial filament, and IND-loaded PVA filaments prepared by HME and IMP. DSC analysis was performed on samples weighing 3 to 5 mg in conventional solid aluminum pans. A temperature program with a range of 20 to 250 °C and a heating rate of 10 °C/min was used. The analysis was carried out at a nitrogen purge rate of 20 mL/min. 

#### 2.3.2. Thermogravimetric Analysis (TGA)

The thermostability of the samples was analyzed by TGA using a simultaneous thermal analyzer (model STA 6000, PerkinElmer, Waltham, MA, USA). About 3 to 5 mg samples were placed in open ceramic pans and heated from 40 to 700 °C at a rate of 10 °C/min in a nitrogen atmosphere (flow rate of 20 mL/min). 

#### 2.3.3. Scanning Electron Microscopy (SEM)

The surface morphology of the filaments and 3D-printed tablets was investigated using SEM microscope (model Mira3, Tescan, Brno, Czech Republic). Samples were placed on aluminum stubs and coated with gold in a vacuum chamber prior to imaging.

#### 2.3.4. Powder X-ray Diffraction (PXRD)

The powder X-ray diffractometer (model MiniFlex II, Rigaku, Tokyo, Japan) was employed for crystallinity investigation of IND in drug-loaded filaments. Samples were analyzed using Cu Kα radiation (λ = 0.154 nm) with a voltage of 30 kV and current of 15 mA. The angular scan in 2-theta range of 5° to 40° with a scan speed of 4°/min.

#### 2.3.5. High Performance Liquid Chromatography (HPLC)

The concentration of IND in the filaments and 3D-printed tablets was analyzed using an HPLC spectrophotometer (model Agilent 1100 series HPLC system, Agilent, Santa Clara, CA, USA). A 20-µL sample solution was injected for analysis using a mobile phase of acetonitrile: methanol: 0.5% v/v phosphoric acid (40:20:40) with isocratic elution throughout the analysis. The sample solution was put into a 150 × 4.6 mm Luna 5u C18 column (Phenomenex, Cheshire, UK). The mobile phase was pumped at a flow rate of 1 mL/min and maintained at 35 °C. The absorbance was detected at a wavelength of 320 nm. All samples were analyzed in triplicate.

#### 2.3.6. Swelling and Matrix Erosion Studies 

The samples (3D-printed tablets using commercial PVA filaments, PVA filaments fabricated by HME, and IND-loaded PVA filaments fabricated by HME) were printed with a cylindrical shape, a diameter of 7.00 mm and a thickness of 2.00 mm. The swelling and matrix erosion studies were adapted from Sriamornsak et al. [16]. Each sample was weighed (W_0_), then placed in a petri dish (90 × 15 mm) with the mesh (weight of mesh, W_m_). Phosphate buffer saline (PBS) pH 6.2 (25 mL) was poured into the petri dish. The petri dishes were placed in a shaker incubator set at 37 ± 0.5 °C and 100 rpm. After 0.5, 1, 2, 4, 6, 8, 10, and 12 h, each sample with mesh was withdrawn from the medium and the excess liquid was subsequently removed using filter paper. The weight (W_1_) was determined. Afterward, the wet samples were dried in a hot air oven at 70 °C for 24 h. Subsequently, each sample was recorded as the final dry weighted value (W_2_). All samples were analyzed in triplicate for each time point. 

The percentage of weight changes, due to absorbed liquid at each time point, was calculated by using Equation (1). The tablet erosion at different time points was estimated using Equation (2).

(1) $$\%\;\mathrm{Weight}\;\mathrm{change}=\frac{W_{1}{-W}_{2}{-W}_{m}}{W_{1}}\;\times \;100$$

(2) $$\%\;\mathrm{Tablet}\;\mathrm{erosion}\frac{W_{0}{-W}_{2}{-W}_{m}}{W_{0}}\;\times \;100$$

#### 2.3.7. Determination of Drug Content

The IND-loaded filaments (100 ± 5 mg), which were taken from three segments of the filaments (both end and center) and 3D-printed tablets containing IND were placed in a volumetric flask with 30 mL solution containing acetonitrile, methanol and 0.5% *v*/*v* phosphoric acid (40:20:40) and sonicated until a clear solution was observed. The solution was then filtered using 0.45-µm filters. The concentration of IND was determined with an HPLC spectrophotometer using the method described in Section 2.3.5. Drug content was calculated using Equation (3).

(3) $$\mathrm{Drug}\;\mathrm{content}\left(\%\;w/w\right)\left(\frac{\mathrm{Weight}\;\mathrm{of}\;\mathrm{drug}\;\left(\mathrm{mg}\right)}{\mathrm{Weight}\;\mathrm{of}\;\mathrm{filament}\;\mathrm{or}\;\mathrm{tablet}\;\left(\mathrm{mg}\right)}\right)\;\times \;100$$

#### 2.3.8. Drug Release Studies

Drug release profiles of IND-loaded PVA filaments and 3D-printed tablets containing IND were obtained using a USP dissolution apparatus I (model AT Xtend™, Sotax, Westborough, MA, USA). The drug release test parameters were used in accordance with the USP 43-NF38 monograph, *“Indomethacin Extended Release Capsules, test 1”* [17]. The tablets were put in a basket and then placed in 750 mL of PBS pH 6.2, at 37 ± 0.5 °C. The rotation speed of the basket was set at 100 rpm. During the release test, 6 mL of each sample solution was withdrawn at various time intervals (i.e., 15, 30, 45, 60 min, 2, 3, 4, 5, 6, 8, 10, 12, 24 h). After that, 6 mL of fresh PBS was added in order to maintain the volume. The solutions were filtered through 0.45-µm filters and the drug concentration was determined with HPLC. 

The drug release kinetics were analyzed using the DDSolver program with Microsoft Excel software (Microsoft Office 365) by fitting the experimental data to mathematical models of drug release using the zero-order, first order, Higuchi, and Korsmeyer–Peppas models. The values of the correlation coefficient (R^2^) were determined to evaluate the best-fit model for the drug release.

## 3. Results and Discussion

### 3.1. Characteristics of Filaments and 3D-Printed Tablets

**Figure 1:**
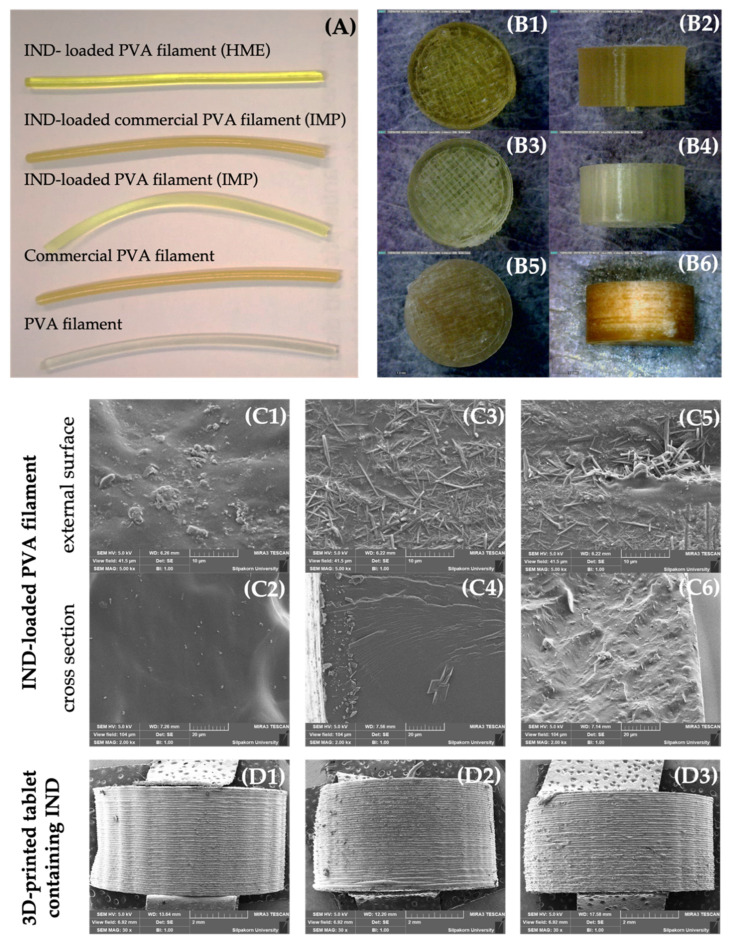
(**A**) Photographs of PVA filaments and IND-loaded PVA filaments fabricated by HME and IMP methods. Top and side view images of 3D-printed tablets containing IND: 3D-printed tablets containing IND, fabricated from (**B1**,**B2**) HME-produced PVA filament, (**B3**,**B4**) IMP-produced PVA filament, and (**B5**,**B6**) IMP-produced commercial PVA filament. SEM images of IND-loaded PVA filaments, fabricated from (**C1**,**C2**) HME-produced PVA filament, (**C3**,**C4**) IMP-produced PVA filament, and (**C5**,**C6**) IMP-produced commercial PVA filament, and SEM images of 3D-printed tablets containing IND, fabricated from (**D1**) HME-produced PVA filament, (**D2**) IMP-produced PVA filament, and (**D3**) IMP-produced commercial PVA filament.

IND-loaded PVA filaments were successfully prepared using the HME process. In addition, the IMP method was compared to the HME method. As shown in Figure 1A, PVA filament extruded from HME at 180 °C was clear with a smooth surface and brittle. Commercial PVA filament had a pale brown opaque color, a smooth surface, and a durable character. The drug-incorporated filament became somewhat brittle when each filament was immersed in saturated drug solution. The IND-loaded PVA filament prepared by the IMP technique for 24 h was bright yellow with a smooth surface. The color of commercial PVA filament loaded with IND by 24-h IMP technique was more faded than that of commercial PVA filament without the IMP process. This finding might be attributed to the deposition of IND on the filament’s surface. The extrudate had a golden hue and a slightly rough surface. This was owing to the fact that water in the filament was continually evaporated when exposed to high temperatures during the HME process [18,19].

The 3D-printed tablets containing IND are depicted in Figure 1(B1–B6); all tablets displayed a grid infill pattern when viewed from the top. The tablet had a thick and smooth coating on the lateral side. Table 1 shows the characteristics of 3D-printed tablets containing IND fabricated from different filaments. The weight, diameter, and thickness of all tablets were similar, except for the drug content. The IND content of tablets produced by the HME process was significantly higher than that of tablets produced by the IMP technique. This implies that when using the IMP technique, part of the drug solution was deposited on the surface of the filament, demonstrating the difficulties in obtaining high drug content from the filament [20].

SEM analysis was used to analyze the morphological attributes of drug-loaded filaments and 3D-printed tablets (Figure 1(C1–C6,D1–D3)). Because of the crystallization of IND in completely melted PVA, filaments made by the HME method had a somewhat smoother surface than filaments prepared by the IMP approach. The surface of IND-loaded filaments produced using the IMP technique had a rough surface. It is assumed that IND were re-crystallized (as needle-shaped crystals) and deposited on the surface of the filament (Figure 1(C3,C5)). The lateral side of the tablets were demonstrated to have consistent overlay layers of filament in all tablets (Figure 1(D1,D3)).

### 3.2. Thermogravimetric Analysis

**Figure 2:**
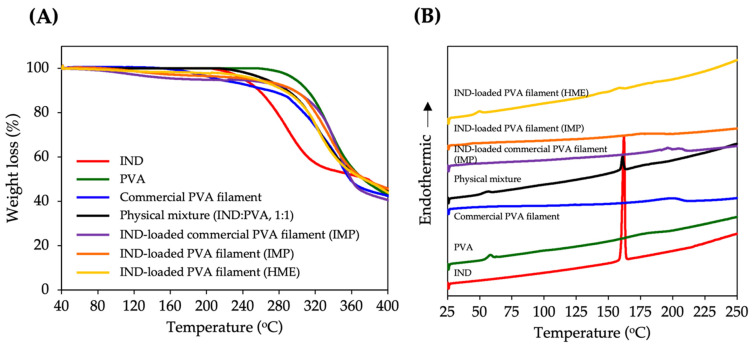
(**A**) TGA thermograms and (**B**) DSC thermograms of IND, PVA, commercial PVA filament, physical mixture, IND-loaded commercial PVA filament (IMP), IND-loaded PVA filament (IMP), and IND-loaded PVA filament (HME).

The TGA thermogram of IND-loaded PVA filament from HME revealed 98.15% of the weight remaining at 180 °C (Figure 2A), implying that IND and PVA remain stable at the filament extrusion temperature (180 °C). According to our preliminary findings [19], TGA thermograms of IND, PVA, and a physical mixture of IND and PVA (1:1) revealed about 5% weight losses at temperatures of 275 °C, 300 °C, and 280 °C, respectively. In this investigation, the required temperature for the printing process was around 190–200 °C. The printing temperature of IND-loaded commercial PVA filament produced using the IMP method was 190 °C. At this temperature, the TGA thermogram of this filament displayed a 5% weight loss, whereas weight losses of 3.45% and 2.18% were detected in the IND-loaded PVA filaments produced by IMP and HME, respectively, at printing temperature (200 °C). Despite the fact that the printing temperature was relatively high (190–200 °C), no significant degradation was observed in the printed tablets. This could be due to the filament’s short resident time inside the printhead [21]. According to TGA results, all filaments had total weight losses of less than 5% at printing temperature, suggesting that these compositions could be used in the FDM 3D printing process.

### 3.3. Differential Scanning Calorimetry

DSC is a thermo-analytical technique used to determine whether a drug has been incorporated into a polymer. Figure 2B depicts the DSC thermograms of all samples. Pure IND showed an endothermic peak at 162.3 °C, indicating its melting point. PVA and PVA filament showed a broad peak in the 178–197 °C range, corresponding to their melting temperatures. At 161.5 °C, a strong endothermic peak was detected for the physical mixture. This result agreed with the thermogram of pure IND. The glass transition temperature (*T_g_*) is defined as the temperature at which polymers lose their elastic characteristics [22], and is an important parameter to consider when analyzing the melting process of a polymer. The polymer becomes sticky and rubbery, just above the *T_g_*, allowing the molecules to attach to the build plate. It was found that IND-loaded PVA filaments fabricated by HME had a *T_g_* of 46 °C, whereas pure PVA had a *T_g_* of 60 °C. The reduction in *T_g_* could be explained by the plasticizing effect of IND. Furthermore, the absence of an endothermic IND peak in IND-loaded PVA filaments indicated that IND has been completely incorporated into the PVA matrix. No endothermic IND peak attributable to the melting of any IND crystal was identified in IND-loaded PVA filaments produced by the IMP technique. These results, however, do not agree with the SEM results, which revealed needle crystals of IND on the surface of the filament. It is assumed that only a few crystals occurred and they could not be probed using DSC.

### 3.4. Powder X-ray Diffraction

**Figure 3:**
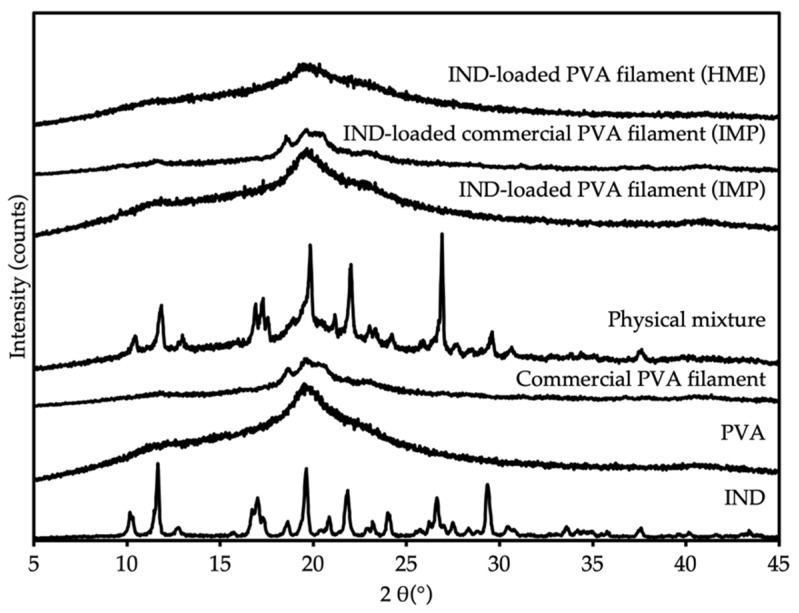
PXRD patterns of IND, PVA, commercial PVA filament, physical mixture, IND-loaded commercial PVA filament (IMP), IND-loaded PVA filament (IMP), and IND-loaded PVA filament (HME).

The crystallinity of IND within filaments was analyzed using PXRD. Figure 3 shows the diffractograms of all samples. The PXRD diffractogram of IND displayed strong crystalline peaks at 10°, 11°, 17°, 19°, 22°, 26° and 29° [23]. The halo peak pattern of pure PVA and commercial PVA filament were given at 19°, describing the semi-crystalline characteristic of PVA [24]. The PXRD pattern of IND-loaded PVA filaments produced by HME showed no peak, suggesting the amorphous nature of the filaments. These findings were consistent with the DSC findings. In the case of IMP-produced filaments, no distinguishing peak was observed in the diffractogram, implying that either the drug was entirely distributed in the polymer matrix or the drug quantity was below the detection limit of the instrument, as previously reported [19].

### 3.5. Swelling and Matrix Erosion Studies

**Figure 4:**
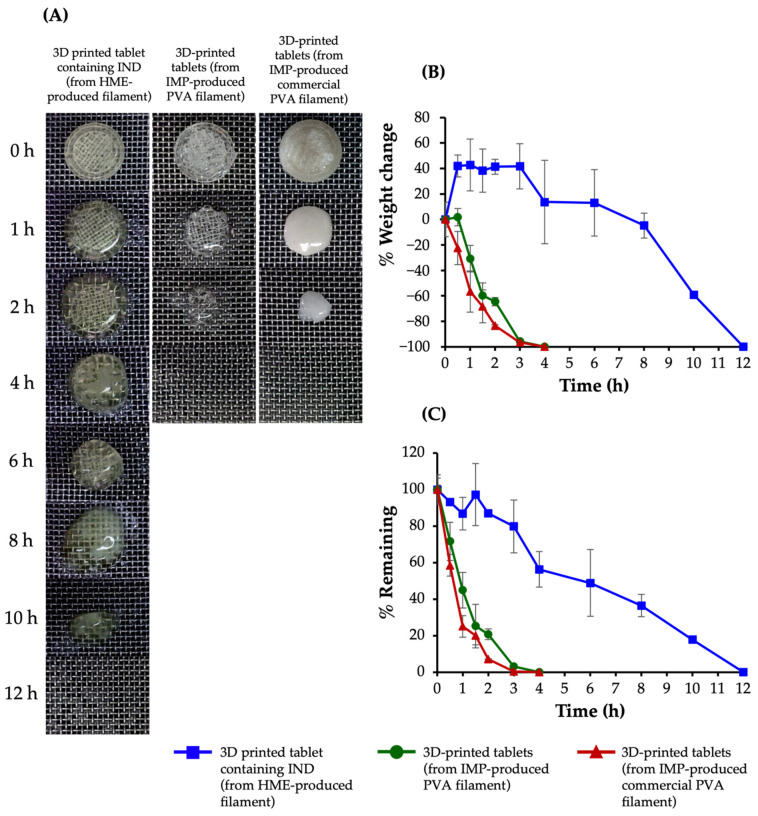
(**A**) Photographs showing swelling behaviors, at various times, of 3D-printed tablets containing IND (from HME-produced filament) and 3D-printed tablets (from IMP-produced PVA filament and IMP-produced commercial PVA filament), (**B**) percentage weight change and (**C**) percentage remaining of 3D-printed tablets containing IND (from HME-produced filament) and 3D-printed tablets (from IMP-produced PVA filament and IMP-produced commercial PVA filament).

Figure 4 depicts the swelling and erosion profiles of 3D-printed tablets containing IND (from HME-produced filament) and 3D-printed tablets (from IMP-produced PVA filament and IMP-produced commercial PVA filament). When the degree of swelling was negative, it indicated that there was a high chance of erosion rather than swelling [25]. For 3D-printed tablets containing IND processed by HME, the swelling ability was roughly 40% within 3 h, and then steadily declined until completely dissolved (within 12 h). Conversely, the swelling profiles of 3D-printed tablets fabricated from IMP-produced PVA filaments and IMP-produced commercial PVA filaments showed a negative value for swelling, suggesting that erosion rather than swelling was the major influence. Furthermore, the tablets eroded in less than 4 h. According to Monschke and Wagner [25], the presence of the drug in the tablets may also influence polymer swelling and erosion. 

### 3.6. Determination of Drug Content

The drug content is an essential factor in determining the characteristics of drug-loaded tablets. The drug loading technique has a considerable influence on the drug content of filaments. The drug content of four IND-loaded PVA filaments including IND-loaded commercial PVA filaments (IMP), IND-loaded PVA filaments (IMP), IND-loaded PVA filaments (HME 5) and IND-loaded PVA filaments (HME 30) was 2.32 ± 0.05, 4.88 ± 0.33, 4.66 ± 0.15 and 28.02 ± 2.34% *w*/*w*, respectively. The IND content of filaments generated by IMP, a post-loading method, was typically low as compared to those produced by HME. The IND content of HME filaments (HME 5 and HME 30) was lower than their initial drug loading, which was most likely due to drug powder adhering to the wall of the filament extruder barrel during the HME process [26]. The drug content of 3D-printed tablets containing IND, on the other hand, was somewhat lower than that of drug-loaded filaments (Table 1). The results showed that IND was perhaps degraded during the 3D printing process. These findings were confirmed by the TGA results. 

### 3.7. Drug Release Studies

**Figure 5:**
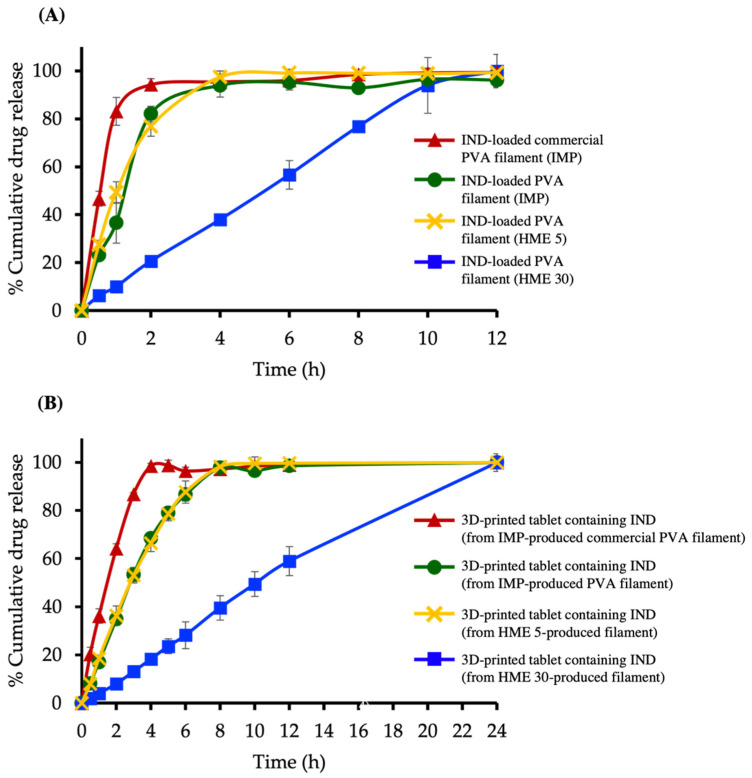
Drug-release profiles of (**A**) IND-loaded PVA filaments and (**B**) 3D-printed tablets containing IND.

Drug release is influenced by many factors, including the method of preparation, drug solubility, drug amount, and drug loading method [27]. Figure 5A depicts the drug release profiles of IND-loaded commercial PVA filaments (IMP), IND-loaded PVA filaments (IMP), IND-loaded PVA filaments (HME 5). and IND-loaded PVA filaments (HME 30). IND-loaded commercial PVA filaments (IMP) and IND-loaded PVA filaments (IMP) were significantly faster, with more than 90% of IND released in 2 h and 4 h, respectively, whereas IND-loaded PVA filaments (HME 5) released 97% in 4 h, which was comparable to the release profile of IND-loaded PVA filaments (IMP). This is most likely owing to the comparable drug content of the two filaments, which was roughly 4.35 and 4.86% *w*/*w*. However, due to their simplicity and flexibility, HME-produced filaments would be more effective. The rate of IND release was slower with high drug loading PVA filament (HME 30) compared with low drug loading PVA filament (HME 5). The drug content impacted the drug release profile of filaments in general. The fastest drug release was seen for the IND-loaded commercial PVA filaments (IMP), which is likely owing to the fact that commercial PVA filaments, unlike pure PVA filaments, may contain a range of components that might impact drug release. 

Figure 5B demonstrates the drug release profiles of 3D-printed tablets containing IND fabricated from four different filaments. The tablets made from IND-loaded commercial PVA filaments (IMP) had a complete drug release within 4 h, whereas the tablets made from IND-loaded PVA filaments (IMP) and low IND loading PVA filament (HME 5) had total drug release in around 8 h. The release from tablets made from the IND-loaded PVA filaments (IMP) released more slowly than the IND-loaded commercial PVA filaments (IMP). This is probably due to a decrease in surface area once the tablet has been formed. During the printing process, the drug on the filament surface was melted and completely embedded into the polymer materials. However, because the drug content of the filaments varied, the drug content in each tablet varied, influencing drug release. IND release from 3D-printed tablets produced from HME filaments with high drug loading (HME 30), on the other hand, increased progressively to 100% within 24 h. It is evident that the drug content had an effect on IND release; for example, IND release from tablets produced from HME 30 was slower than that from tablets made from HME 5. When comparing fabrication methods, no difference in release profile was observed. In general, drug release from 3D-printed tablets is affected by surface area [28], infill density, infill percentage [29], and carrier material properties [13]. In the current study, the form, size, infill density, and infill percentage of the 3D-printed tablets containing IND were all fixed. The only variables that changed were the drug content [30] and the source of the carrier material. As a result, drug release was entirely reliant on these factors.

The 3D-printed tablets with different drug loading methods undergo different drug release mechanisms. Because the tablets were composed of PVA as the carrier material, the erosion behavior of the PVA should be dominant. The swelling study, on the other hand, suggested that tablets made from HME-produced filaments may undergo swelling followed by erosion. Therefore, the overall drug release behaviors of the tablets made from HME-produced filaments differ from those made from IMP-produced filaments. To interpret the drug release behaviors of the 3D-printed tablets, mathematical models with consideration of one or more of the characteristic drug release behaviors were used, including the zero-order model, first-order model, Higuchi model, and Korsmeyer–Peppas model. All filaments and tablets had the greatest R^2^ values in the Korsmeyer–Peppas release model, as shown in Table 2. The Korsmeyer–Peppas model is used to describe drug release from polymeric systems. The mechanism of drug release from cylindrical tablets is explained by the release exponent (*n*) value in this model, *n* = 0.45 for Fickian diffusion. A value of *n* between 0.45 and 0.89 indicates non-Fickian diffusion (anomalous transport). Case I transport is defined as zero-order release kinetics when *n* = 0.89. Another scenario is the *n* > 0.89 corresponding super case II transport [31,32]. 

The *n* values for the filaments produced from commercial PVA filament (IMP) and PVA filament (HME 5) were 0.83 and 0.71, respectively, suggesting non-Fickian diffusion. PVA filament (IMP) had an *n* value of 1.02 while PVA filament (HME 30) had an *n* value of 0.97. The kinetic was assumed to be super case II transport. According to the SEM image (Figure 1(C3)), the drug deposited on the surface of PVA filaments produced by IMP diffused and eventually dissolved in the medium. In case of 3D-printed tablets made from IMP filaments, an *n* value ranging from 0.45 to 0.89 suggested that the drug release was non-Fickian diffusion or anomalous transport, which was regulated by matrix erosion and drug diffusion [16]. On the contrary, the release exponent of 3D-printed tablets made from HME filament was more than 0.89 (super case II transport), indicating that the drug was released by matrix polymer diffusion and relaxation [33]. This observation was consistent with a swelling and erosion study of 3D-printed tablets made from HME filament, which revealed that the drug was released through a swellable polymeric matrix and an erosion mechanism.

## 4. Conclusions

The 3D-printed tablets containing IND made from drug-loaded PVA filaments were fabricated by HME and IMP methods. The IND-loaded filaments produced by IMP and HME with comparable drug content demonstrated a consistent drug release profile. Furthermore, drug release from 3D-printed tablets containing IND using IMP-produced filaments and HME-produced filaments with low drug loading (HME 5) was faster, confirming that the mechanism was a polymeric matrix erosion mechanism. The drug release of 3D-printed tablets made from HME-produced filaments with high drug loading (HME 30) was extended to 24 h, with a swelling-erosion mechanism. This study clearly demonstrated that the drug loading technique has a considerable impact on drug content, which in turn has a big impact on drug release from 3D-printed tablets. This may be used to develop dosage forms for personalized medicine using FDM 3D printing. 

## Figures and Tables

**Table 1:** Characteristics of 3D-printed tablets containing IND fabricated from different filaments.

Filament Used	Weight (mg)	Diameter (mm)	Thickness (mm)	IND Content (%)
Commercial PVA filaments (IMP)	153.61 ± 0.46	7.11 ± 0.23	4.23 ± 0.01	1.44 ± 0.02
PVA filaments (IMP)	139.54 ± 4.42	6.98 ± 0.02	3.59 ± 0.16	4.86 ± 0.26
PVA filaments (HME 5) *	151.39 ± 1.86	7.07 ± 0.02	4.03 ± 0.04	4.35 ± 0.22
PVA filaments (HME 30) *	158.07 ± 0.05	7.01 ± 0.15	4.32 ± 0.20	27.94 ± 1.43

* HME 5 and HME 30 are PVA filaments loaded with 5% *w*/*w* and 30% *w*/*w* of IND, produced by HME.

**Table 2:** Release kinetic values of IND from IND-loaded filaments and 3D-printed tablets containing IND prepared by using different filaments, fitted to the best-fit release kinetic model.

Sample	Zero-Order	First-Order	Higuchi	Korsmeyer–Peppas	
	(R^2^)	(R^2^)	(R^2^)	(R^2^)	(*n*)
Filaments containing IND					
Commercial PVA filaments (IMP)	0.9939	0.9811	0.9720	1.0000	0.83
PVA filaments (IMP)	0.9934	0.9369	0.8745	0.9936	1.02
PVA filaments (HME 5)	0.9547	0.9978	0.9695	0.9981	0.71
PVA filaments (HME 30)	0.9989	0.9722	0.8785	0.9992	0.97
3D-printed tablets containing IND					
Commercial PVA filaments (IMP)	0.9532	0.9808	0.9547	0.9946	0.72
PVA filaments (IMP)	0.9411	0.9229	0.9297	0.9824	0.74
PVA filaments (HME 5)	0.9440	0.9758	0.9355	0.9866	0.74
PVA filaments (HME 30)	0.9881	0.9130	0.8520	0.9926	0.91

## Data Availability

Data is contained within the article.
